# CAPMH: development of the first open access journal in the field of child mental health

**DOI:** 10.1186/s13034-021-00426-x

**Published:** 2021-11-30

**Authors:** Andreas Witt, Gerrit van Schalkwyk, Jörg M. Fegert

**Affiliations:** 1 Department of Child and Adolescent Psychiatry and Psychotherapy, University of Ulm, Steinhövelstr. 1, 89075 Ulm, Germany; 2 Department of Pediatrics, University of Utah, Salt Lake City, USA

Child and Adolescent Psychiatry and Mental Health (CAPMH) was founded in 2007, an initiative of Prof. Fegert. He served as the Editor-in-Chief, with support from Dr. Benedetto Vitiello (Italy) as Deputy Editor-in-Chief and Prof. Goldbeck (Germany) and Jacinta Tan (UK) as Associate Editors. The journal was the first independent, open access, online journal in the field with the mission to provide an international platform for rapid and comprehensive scientific communication on child and adolescent mental health issues from diverse cultures and contexts.

The first issue of CAPMH was released in 2007. In February 2013, CAPMH became the official journal of the International Association for Child and Adolescent Psychiatry and Allied Professions (IACAPAP) and has later also been affiliated with the European Association for Forensic Child and Adolescent Psychiatry, Psychology and other involved Professions (EFCAP). Since its inception, the journal has grown rapidly and has received funding by different foundations. However, the journal also faced difficulties and setbacks as sadly, Prof. Goldbeck, one of the founding editors, unexpectedly passed away in 2017.

To further establish the journal in the field of child mental health, the editorial work assertively to establish an impact factor. CAPMH was under consideration to receive an impact factor in 2009 and 2011. Finally, an impact factor was received in 2015. The current impact factor for 2020 is 3.033—a remarkable achievement given the young age and broad mission of the journal. With the new impact factor, the journal has climbed up to rank 37 in Pediatrics, ranks 91 in Psychiatry (Science Citation Index Expanded, SCIE) and remains at rank 66 in Psychiatry (Social Science Citation Index, SSCI), underlining the importance of the journal in the field of child mental health.

The growth of the journal is also reflected in the number of submissions received. Most notably, a dramatic increase in submissions was observed after the journal was awarded an impact factor. Another indicator for the success of the journal is the number of downloads of published articles. With now almost 8,00,000 downloads per year, scholars and practitioners publishing within the journal reach a vast readership worldwide.

Since its foundation the aim of the journal has been to increase the knowledge base related to the diagnosis, prognosis and treatment of mental health conditions in children and adolescents, and to integrate basic science, clinical research and the practical implementation of research findings [1]. Further, the journal offers a platform for reporting factors and mechanisms that help children and adolescents to maintain their mental health. As such, the journal is a rich venue for a multidisciplinary audience; this includes psychiatrists, pediatricians, psychologists, neuroscientists, and allied disciplines.

One outstanding attribute of CAPMH as the first worldwide open access journal in the field of child and adolescent psychiatry, is its international focus. This is reflected by the international editorial board, submissions, publications, and accesses from all over the world. Thematic series focusing on child and adolescent psychiatry in Africa [2] and Asia [3] underline this international focus. The editors particularly encourage authors from countries less represented in the child and adolescent psychiatry literature to submit their work.

Besides the publication of incoming manuscripts, CAPMH has focused on specific topics in child and adolescent psychiatry throughout the years. The latest thematic series focuses on the impact of the Covid-19 pandemic [4] and is currently still accepting manuscripts. Prior thematic series focused on forensic child and adolescent psychiatry and mental health [5–8], Non-suicidal self-injury [9–11], child abuse and neglect [12], e-Learning [13], identity [14], early interventions [15] and psychopharmacology [16].

The success of the journal would not have been possible without the work of the committed editors, the editorial board and reviewers. In November 2021 the journal has undergone a new transition as Prof. Fegert stepped down as from his role as Editor-in-Chief and Dr. van Schalkwyk and Dr. Witt have assumed the role together. The new Editors-in-Chief want to express their deep gratitude to the founding editors of Child and Adolescent Psychiatry and Mental Health, who laid the foundation for this exciting project. They want to especially thank Prof. Fegert who has led and shaped the journal since its foundation in 2007. With his work he profoundly contributed to the reach and success of the journal and as the founding editor he stays associated with the editorial team Their thanks also go out to all editors who have been and are currently working with the journal, as well as the editorial board and the vast number of reviewers who have supported the journal throughout the years.

The aim of the new Editors-in-Chief over the next years to come is a continuation of the excellent work of Child and Adolescent Psychiatry and Mental Health and to further advance the impact of the journal in the field. As the flagship journal of the International Association of Child and Adolescent Psychiatry and Allied Professions (IACAPAP) the Editors-in-Chief seek to provide a scientific platform for high quality research and practice in this area by ensuring a rigorous and timely review process. To ensure a timely and quality review process, the aim is to build a reliable and committed board of reviewers that form a relationship with the journal. To further increase the impact of the journal in the field, the aim is to establish thematic series on a regular basis. Such thematic series can address pressing issues of the time and be edited by guest experts or by editors of the journal.

As an international journal for the area of child and adolescent psychiatry and mental health, the vision of the new Editors-in-Chief is to create an international scientific platform that addresses all professions in mental health. As the journal should reflect this international focus, the aim is to increase diversity among the editorial board not only in regards to gender and ethnicity, but also concerning professional backgrounds, methodological and scientific orientation. Therefore, the aim of CAPMH is to attract researchers from all over the world to submit their work to the journal not only from developed countries but also to support high quality work from less developed countries to further advance science and practice in the field of child and adolescent psychiatry and mental health.

## Data Availability

Not applicable.
